# 12-*O*-tetradecanoylphorbol-13-acetate (TPA) increases murine intestinal crypt stem cell survival following radiation injury

**DOI:** 10.18632/oncotarget.17269

**Published:** 2017-04-20

**Authors:** Yaojie Liang, Hongwei Zhou, Yibing Yao, Ailing Deng, Zhihong Wang, Boning Gao, Minhang Zhou, Yu Cui, Lili Wang, Lei Zhou, Bianhong Wang, Li Wang, Anqi Liu, Lanlan Qiu, Kun Qian, Yejian Lu, Wanping Deng, Xi Zheng, Zhengtao Han, Yonghui Li, Junzhong Sun

**Affiliations:** ^1^ Department of Geriatric Oncology, The First Affiliated Hospital of Chinese PLA General Hospital, Beijing, China; ^2^ Department of Hematology, The First Affiliated Hospital of Soochow University, Suzhou, China; ^3^ Hamon Center for Therapeutic Oncology Research, UT Southwestern Medical Center, Dallas, Texas, USA; ^4^ State Key Laboratory of Proteomics, Beijing Proteome Research Center, Beijing Institute of Radiation Medicine, Collaborative Innovation Center for Cancer Medicine, Beijing, China; ^5^ Department of Hematology, Chinese PLA General Hospital, Beijing, China; ^6^ Department of Hematology, Beijing Tsinghua Changgung Hospital, Tsinghua University, Beijing, China; ^7^ Department of Hematology, Laoshan Branch, No.401 Hospital of Chinese PLA, Qingdao, China; ^8^ Department of Critical Care Medicine, Beijing Electric Power Hospital, Capital Medical University, Beijing, China; ^9^ Department of Hematology, Beijing Shijitan Hospital, Capital Medical University, Beijing, China; ^10^ Henan Tumor Research Institute, Zheng Zhou, China

**Keywords:** 12-O-tetradecanoylphorbol-13-acetate (TPA), intestinal crypt stem cells, radiation injury, IEC-6 cell and BALB/c mouse, radioprotection

## Abstract

Radiation enteropathy is a common complication in cancer patients following radiation therapy. Thus, there is a need for agents that can protect the intestinal epithelium against radiation. 12-*O*-tetradecanoylphorbol-13-acetate (TPA) has been shown to induce differentiation and/or apoptosis in multiple cell lines and primary cells. In the current report, we studied the function of TPA in radiation induced enteropathy in cultured rat intestinal epithelial cell line IEC-6 after ionizing radiation (IR) and in mice after high dose total-body gamma-IR (TBI). In IEC-6 cells, there were reduced apoptosis and cell cycle arrest in TPA treated cells after IR. We detected a four-fold increase in crypt cell survival and a two-fold increase in animal survival post TBI in TPA treated mice. The beneficial effects of TPA were accompanied by upregulation of stem cells markers and higher level of proteins that are involved in PKC signaling pathway. In addition, TPA also decreased the TBI-augmented levels of the DNA damage indicators. The effects were only observed when TPA was given before irradiation. These results suggest that TPA has the ability to modulate intestinal crypt stem cells survival and this may represent a promising countermeasure against radiation induced enteropathy.

## INTRODUCTION

Radiotherapy is one of the major treatment modalities used to control or eradicate malignant solid tumors, which is used in at least 50% of patients with cancer and plays a crucial role in 25% of cancer cures [1]. The intestinal epithelium is very sensitive to ionizing radiation (IR) injury [2, 3], and is one of the organs most vulnerable to radiation toxicity [4]. Radiation enteropathy is a common complication in cancer patients following radiotherapy, especially in those treated for abdominal and pelvic tumors who experience more pronounced side effects than others. Gastrointestinal(GI) mucositis manifests with symptoms, which include: nausea and vomiting, pain, diarrhea, constipation and rectal hemorrhage [5]. In addition, nuclear accidents lead to risk of radiation exposure, which can cause radiation-induced injury. Therefore, effective therapeutic remedies are urgently needed, and identifying effective and useful substances for the prevention or treatment of intestinal and others injuries due to radiation exposure is of utmost importance.

Radiation-induced enteropathy is a complex pathophysiological process. Acute intestinal injury results from inflammation and direct cell death in the rapidly proliferating crypt epithelium, in which lead to insufficient replacement of the villus epithelium and progressive breakdown of the mucosal barrier. Epithelial injury and diarrhea, which considerably contribute to early radiation-induced morbidity and mortality, are closely linked to endothelial apoptosis and vascular dysfunction, in which cause the transfer of intravascular fluids to the gut lumen [6, 7]. Exposure to high doses of total body radiation (≥9 Gy) triggers an acute GI radiation toxicity syndrome (GI toxicity) that often leads to death, regardless of intervention with advanced therapeutics or bone marrow transplants [8, 9]. The high mortality associated with GI toxicity is thought to result from the radiation-induced damage to the intestinal and colonic mucosa, which leads to decreased fluid absorption, electrolyte imbalance, impaired barrier function, bacterial translocation, and organ failure [10–12]. This sequence of conditions in the GI system is initiated by radiation-induced damage to stem cells that must continually proliferate to maintain the integrity of the crypt and its regeneration [13, 14]. Crypt cells in both the small intestine and colon are prone to radiation damage and serve as a marker of potential survival following total body radiation [15]. Accordingly, it is necessary to develop novel therapeutic drugs that can prevent damage to GI crypt stem cells in order to increase survival [16]. To date, only a few mitigating or radioprotective agents have been approved by the FDA. Most of them are only effective in treating the hematopoietic syndrome triggered by low dose radiation exposure, and fail in the treatment of GI toxicity induced by high-dose radiation exposures, for the thousands of potentially exposed individuals [17].

Croton tiglium L, a leafy shrub of the Euphor-biaceae family, is native to Southeastern Asia. 12–*O*–tetradecanoylphorbol-13-acetate (TPA, also known as PMA), the main active constituent of croton oil, is an irritant and inflammatory agent that has been widely used as a tumor promoter [18, 19]. TPA has been demonstrated to modulate the growth, differentiation, survival, function, and metabolism of a variety of primary cells and cell lines [20–23]. The function of TPA in the protection of radiation induced apoptosis in endothelial cells has been shown [24]. In the first report of administration of TPA to humans, Zhengtao Han et al. [18, 25] treated 12 patients with myelocytic leukemia, who were refractory to other drugs, with various small doses of TPA for various length of times, and found that several of the patients treated with combination therapy or TPA alone exhibited a decrease in the number of leukemic cells in blood or bone marrow with criteria for complete or partial remission being documented in 7 patients. This study was the first to show the good therapeutic effect of TPA. The function of TPA in the protection of radiation induced enteropathy has not been reported. In this study, we examined the protective effect of TPA pretreatment against intestinal injury in a rat intestinal epithelium cell line IEC-6 and in a murine model of intestinal damage after IR.

## RESULTS

### TPA mitigated radiation-induced cell apoptosis and cell cycle arrest and increased proliferation of IEC-6 cells following γ-IR

**Figure 1:**
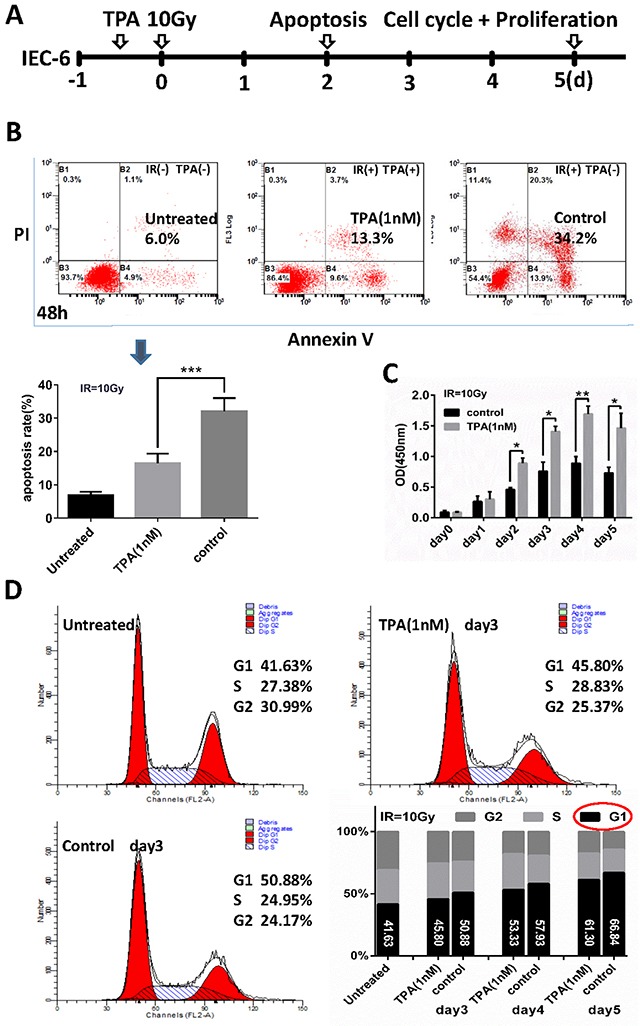
Effects of TPA on radiation-treated cells *in-vitro* **(A)** Schematic representation of the vitro experimental design. IEC-6 cells were treated with TPA for 12 h before radiation and apoptosis, cell proliferation and cell cycle analysis were detected 48 h, 0-5 days and 3-5 days after IR respectively. **(B)** Scatterplots of apoptosis rates in IEC-6 cells without IR treatment (Normal, left panel), cells exposed to IR with 1nM TPA treatment (middle panel) or without TPA treatment (right panel). Summary of the apoptosis rate in the three groups is shown in the bar graph. **(C)** Effect of TPA on the IEC-6 cell proliferation was determined with the Cell Counting Kit-8 assay. **(D)** The percentage of cells in each phase was determined by flow cytometric analysis. Mean ± SEM. *P ≤0.05; **P ≤0.01.

The vitro experimental design is schematically presented in Figure 1A. To delineate its role in cell survival, we evaluated whether TPA altered apoptosis following γ-IR exposure. We treated IEC-6 cells with 1nM TPA for 12h before radiation, cells were doubly-stained with propidium iodide and annexin V–FITC, and analyzed them by flow cytometry. At 48 hours, cells in the TPA-treated group had a lower apoptosis rate (16.53% ± 1.41%) than did control group cells (32.05% ± 2.00%), although the apoptosis rates under these two conditions are higher than the untreated cells (6.80% ±0.80%) as shown in Figure 1B. We also treated IEC-6 cells with 1nM TPA for 12h before irradiation, stained them with cell counting kit-8 to assess their proliferation. The proliferation of the TPA-treated IEC-6 cells was gradually increased in the initial 4 days. Meanwhile, the proliferation of the control non-TPA treated IEC-6 cells was also increased, but at a lower rate than the TPA-treated group as shown in Figure 1C. In addition, the flow cytometric analysis, revealed that radiation induced cell cycle arrest at G1 phase while TPA promoted the transition from G1 to S phase of the cell cycle. These findings suggested a significant proliferative effect of TPA on cell cycle progression following radiation exposure (Figure 1D).

### TPA increased crypt survival in BALB/c mice following γ-IR

**Figure 2:**
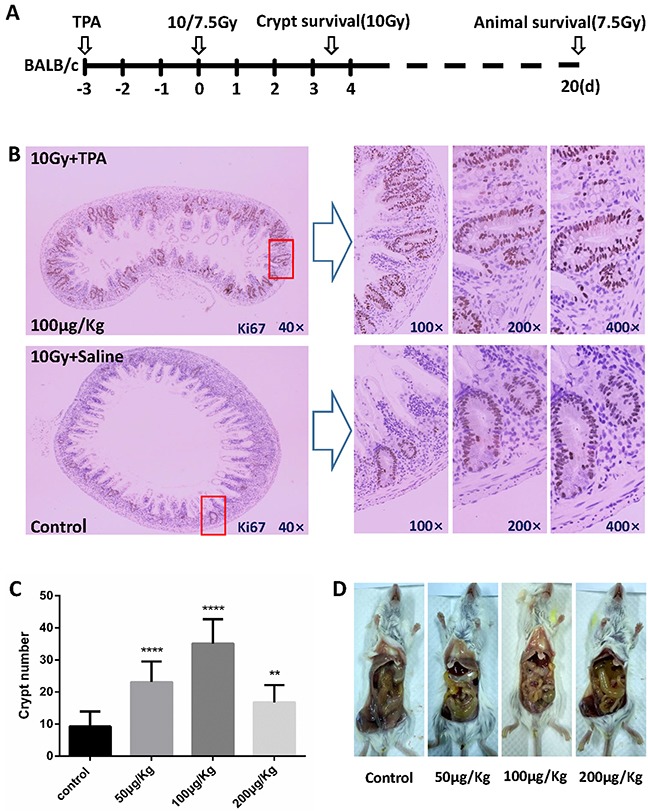
Effects of TPA on crypt survival after radiation exposure **(A)** Schematic representation of the vivo experimental design. BALB/c mice were treated with Saline or TPA via tail vein for 3 days prior to 10Gy TBI (n = 3 in each group) and were euthanized at 3.5 days after the IR for crypt survival assays. Another set of mice were subjected to overall animal survival studies for up to 20 days post-IR, and times of death were noted. **(B)** BALB/c mice in the control group or the TPA-treated group were subjected to TBI (n = 3 in each group). These mice were euthanized 3.5 days post-IR. The small intestines were harvested, fixed, and processed. The presence of more than five Ki67 positive cells grouped together was recorded as surviving crypts. Representative images (40×magnification) of intact small intestine crypts harvested at 3.5 days post-IR from mice treated with either saline or TPA. **(C)** Average number of surviving crypts per cross section was counted and presented. The values in the bar graph are given as average ± SEM and denote statistically significant differences (*P*<0.0001) compared to control. **(D)** Edema and synechia of small intestine were observed by gross anatomy observation in each group.

The vivo experimental design is schematically presented in Figure 2A. To further investigate the effects of TPA on radiation-induced crypt cell death, TPA was injected daily into BALB/c mice via tail vein for 3 days before subjecting them to 10Gy TBI. After the IR treatment, animals were euthanized on day 3.5, the whole intestine was fixed and the distal jejunum was immunostained with Ki67 antibodies. The surviving crypt cells were identified as Ki67 positive cells. There was an average of 9.29 surviving crypts per cross section in the control group (Figure 2B, 2C). In comparison, in the pre-IR TPA-treated group, a 3.78-fold increase in surviving crypts (35.09 crypts) per cross section (Figure 2B, 2C) was detected. There was no difference in the number of surviving crypts between the post-IR TPA treated and control animals (data not shown). Pictures of intestine after different treatment are shown to demonstrate the effect of TPA after radiation. As shown in Figure 2D, small intestines in all groups suffered damage including wall thinning and edema. However, the damage was the least in the pre-IR TPA treatment (100μg/Kg) group compared with the control and 50μg/Kg, 200 μg/Kg TPA treatment group.

### TPA prolonged animal survival and reduced weight loss in BALB/c mice following γ-IR

**Figure 3:**
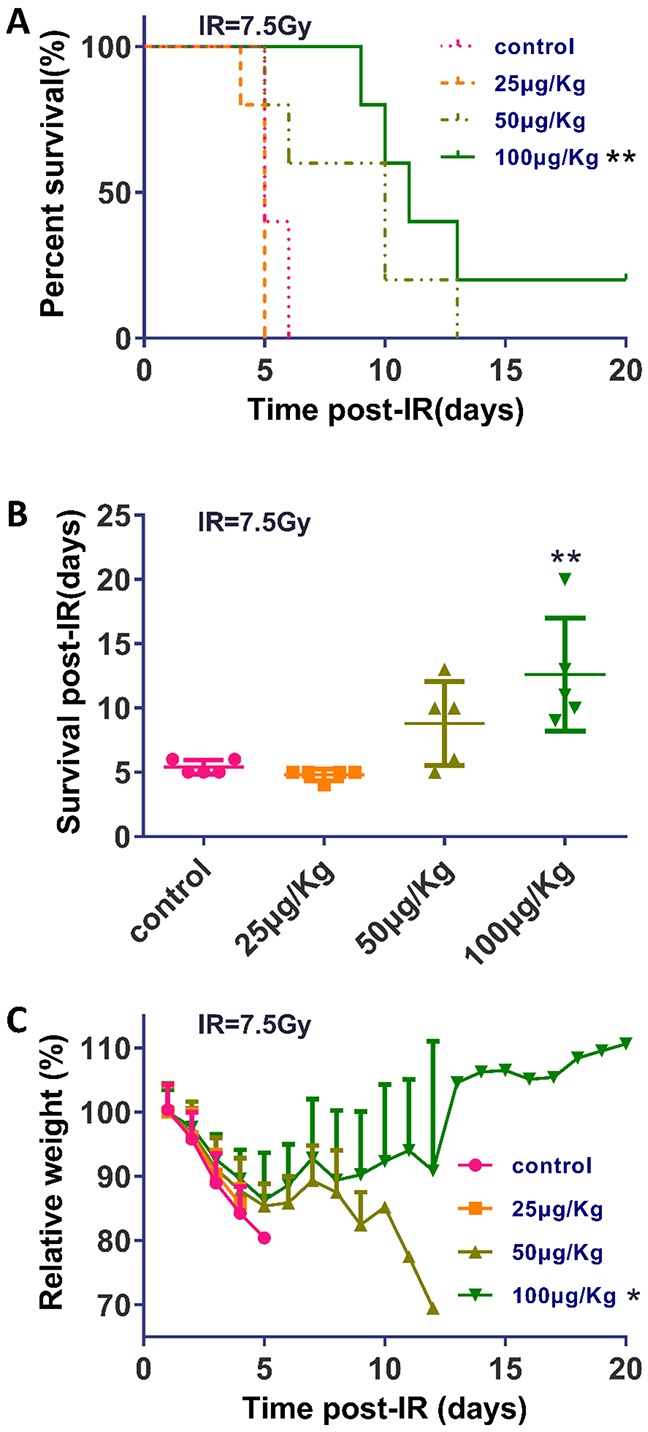
TPA prolonged survival and reduced weight loss in mice following γ-IR **(A)** Graphic illustration of the percent survival of mice (n = 5 in each group) that underwent the indicated treatments. **(B)** Scatter plot graph depicting the day of death of mice that underwent the indicated treatments. **(C)** Mice were weighed at the indicated times after γ-IR. Weight measurements are shown in percent of the initial weight. Mean ± SEM. *P ≤0.05; **P ≤0.01 (100 μg/Kg vs control).

To assess the effects of the TPA concentration on mice survival, we performed experiments with different doses of TPA. BALB/c mice were subjected to 7.5Gy (dose rate 100.2cGy/min) TBI with different dose TPA treatment or no TPA treatment (control). The control group and the lowest dose (25μg/Kg) of TPA treated group survived an average of 5.4 days (with a median survival of 5 days) after TBI, whereas higher doses of TPA treated mice survived an average of 8.8 (50μg/Kg, median survival of 10 days) and 12.6 days (100μg/Kg, median survival of 11 days) respectively (Figure 3A, 3B). The difference is statistically significant (log rank p = 0.0023) between the survival of the control and TPA 100 μg/Kg treated groups. Starting on day 1 post-IR, there was a weight loss in all groups. However, the mean body weight loss was lower in the TPA groups than in the control group. Moreover, the body weight of the mice in the 100 μg/Kg TPA-pretreated group began to recover by day 5 post-radiation (Figure 3C).

### Increased expression of intestinal stem cell genes in the small intestine and proteins involved in PKC signaling pathway in IEC-6 cells, decreased the levels of the DNA damage indicators following TPA pretreatment after γ-IR

**Figure 4:**
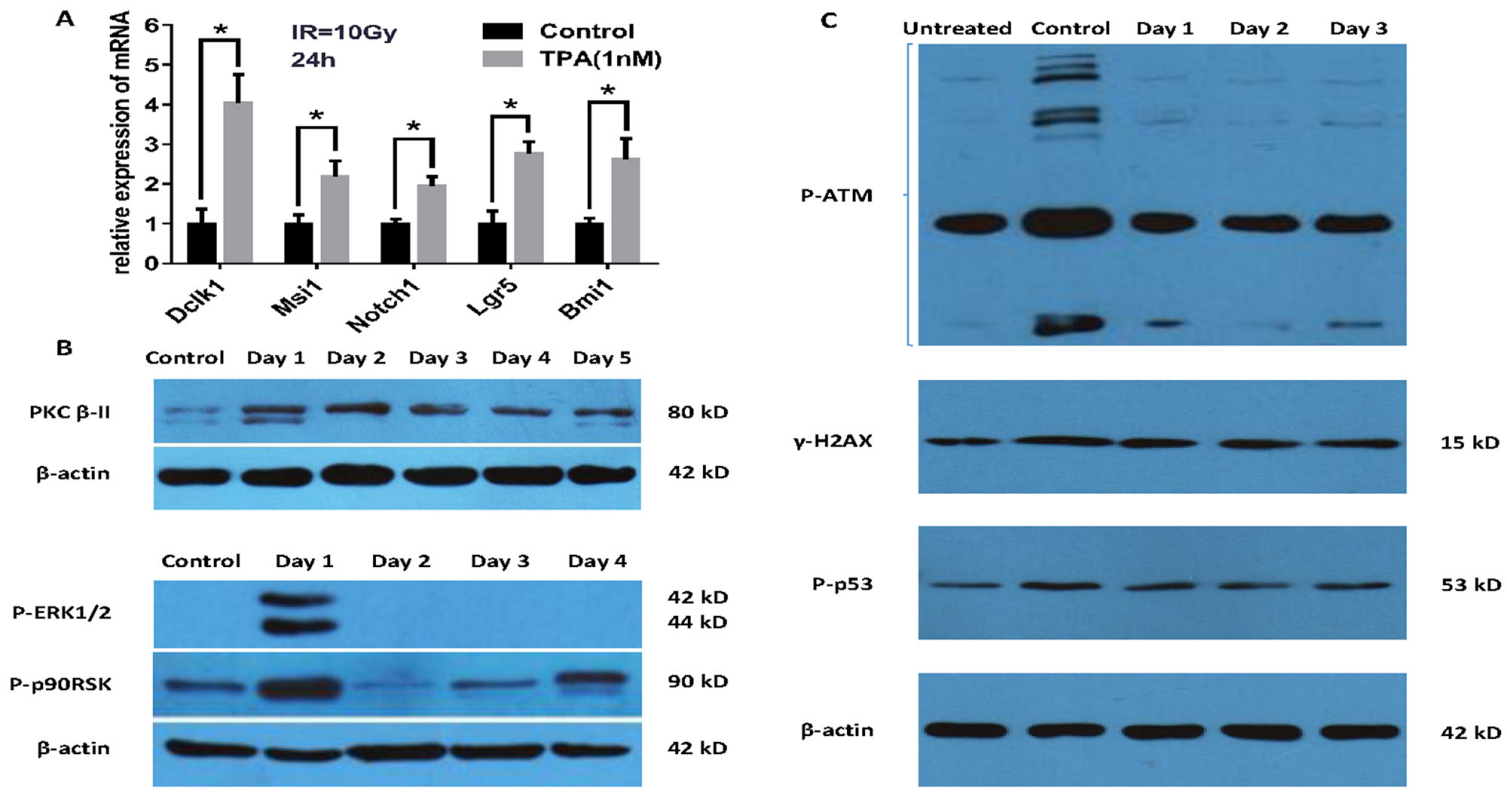
Study on the mechanism of TPA as a radioprotective agent **(A)** TPA increases intestinal stem cell genes expression 24 h post-IR. **(B)** Verification of the key signaling pathway proteins of the putative model by Western blot analysis. TPA activates PKC-βII, ERK1/2 and p90RSK phosphorylation. **(C)** TPA concomitantly reduces P-ATM, γH2AX and P-p53 levels at day1 and day2 following the pretreatment after γ-IR. Mean ± SEM. *P ≤0.05.

We investigated the mechanisms involved in the mitigation of the radiation effects by TPA in vitro and vivo. After 24 hours post-IR, total RNAs from the small intestine of BALB/c treated by TPA or not were subjected to qRT-PCR analysis. We detected a significant upregulation of *Dclk1* (4.03-fold), *Msi1* (2.17-fold) and *Notch1* (1.93-fold) in IEC-6 cells pretreated with TPA compared with the control (IR but no TPA treatment) cells. Noteworthy,*Msi1* is an intestinal stem/progenitor cell marker. Moreover, we also observed increased expression of the putative ISCs markers *Lgr5* (2.76-fold) and *Bmi1* (2.61-fold) in the TPA group (Figure 4A). Additionally, we observed that the protein levels of activated PKC-βII, P-ERK1/2 and P-p90RSK, which are involed in PKC signaling pathway, were increased at day 1 in TPA treated cells (Figure 4B). TPA also decreased the levels of the DNA damage indicators P-p53, γH2AX and P-ATM at day1 and day2 following TPA pretreatment after γ-IR (Figure 4C).

## DISCUSSION

The increased probability of a nuclear and radiation accident and incident in the world has led to a search for effective countermeasures capable of diminishing the potentially lethal effects of radiation on bone marrow, vital organs, and the GI tract [16, 26]. The GI and hematopoietic systems are highly sensitive to radiation, and the GI syndrome and myelosuppression are major causes of death following radiation exposure [27]. In general, rapidly dividing cells, such as intestinal-mucosa and bone marrow cells, are most vulnerable to radiation. Indeed, 60-80% of patients experience temporary symptoms of bowel toxicity during radiation therapy. Moreover, 50% of patients who have undergone abdominal or pelvic radiation therapy suffer from some degree of chronic intestinal dysfunction, and radiation enteropathy is one of the most common side effects among long term cancer survivors [1].

Acute, whole-body doses of radiation cause a gastrointestinal syndrome, primarily as a result of the death of intestinal mucosal stem cells. [4]. The free radical scavenger, amifostine, is the conventional drug currently approved for reduction of the side effects of radiation therapy. While amifostine has shown impressive effects in some animal studies, and has also shown some effect in preventing clinical GI radiation toxicity, serious side effects from this drug and a narrow therapeutic time window severely limits its use [1]. Accordingly, the development of radioprotective agents with low toxicity and an prolonged window of protection has attracted a great deal of attention.

Intestinal crypt stem cells, located at the base of the intestinal crypt, maintain a strong ability of proliferation and differentiation and a dynamic balance. Radiation causes the death of intestinal crypt epithelial cells and as a result crypt stem cells cannot be timely replaced [28]. However, intestinal crypt cell count at 3.5 days after the radiation is an important index to evaluate the crypt stem cell regeneration [29]. Thus, it has become an important reference index to evaluate the effect of radioprotective agents.

In recent years, with the continuous deepening of the study of ISCs, a more profound understanding of its characteristics is being achieved. For instance, N. Barker et al found that [30] the Wnt signaling pathway regulating the downstream gene *Lgr5* is activated in small intestinal crypt at the bottom of the Paneth cells between the crypt base columnar cells. Inducible Cre knock-in allele and the Rosa26-lacZ reporter strain, lineage-tracing experiments confirmed that Lgr5-positive crypt base columnar cells generated all the epithelial lineages over a 60-day period, suggesting that it represents the stem cell of the small intestine and colon. As a result, much attention has been devoted to the intestinal stem cell population over the last few years. Currently at least four types of intestinal stem cell markers have been identified, namely *Bmi1* [31], *Dclk1* [32], *Msi1* [33], *Notch1* [32].

**Figure 5:**
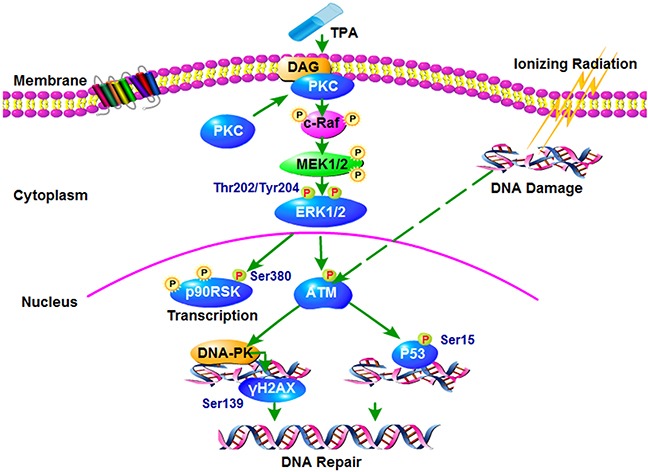
Proposed working model for the signaling pathways that mediates the radiation-induced injury It indicates TPA may represent a promising countermeasure against radiation induced enteropathy and warrants further studies in its role as radioprotective agent.

We present here the possible anti-radiation signaling pathway of TPA, specifically, the PKC-ERK1/2-TAM pathway (Figure 5). The expression of the key signaling pathway proteins of this pathway, were validated by Western blot analysis (Figure 4B, 4C).

The evidence suggest that Protein kinase C (PKC) does not promote cancer development; on the contrary, PKC isozymes generally function as tumor suppressors and can inhibit the growth of tumors [34]. The reason why inhibiting PKC failed in the clinic is that prolonged or repetitive treatment with phorbol esters (TPA) depletes the cPKC and nPKC isozymes in the cells, which brings into question whether loss of PKC, rather than its activation, promotes tumorigenesis [35]. The extracellular signal regulated kinase (ERK1/2) is an important cell signaling protein, which can transfer the extracellular information to the nucleus, and elicit the ultimate reaction in the cell. Several studies have shown that ERK1/2 is an important downstream active molecule of PKC [36, 37]. Additionally, the *ATM* gene is a member of the PI-3K family, and its encoded protein can regulate DNA repair and adjust the function of the cell cycle, and in this way it can activate DNA repair genes, such as *DNA dependent protein kinase (DNA-PK)* and *p53* after radiation. Other studies showed that after radiation, ERK1/2 can activate ATM against radiation injury [38].

Among the proteins participating in this pathway, DNA-PK is the key protein kinase involved in the DNA damage repair process, which determines the whole process of the DNA damage repair pathway [39]. In DNA damage, DNA double strand breaks are the most lethal, repair is mainly through the DNA-PK led DNA non homologous end connection [40]. DNA-PK can activate γH2AX and p53, and starts DNA repair. γH2AX can activate the DNA damage repair system and performs a repair function [38, 41]. p53 is an important molecule for the control of acute small bowel damage after irradiation [42–44], which can also activate the downstream proteins to perform DNA damage repair and anti-radiation function [45]. In addition, we found that the P90RSK protein is also activated, and this protein kinase participates in many biological functions. In fact, it is an important signal protein that regulates cell growth, proliferation, cell survival and cell migration [46].

## MATERIALS AND METHODS

### Cell culture and reagents

Rat intestinal epithelium cells (IEC-6) were purchased from the American Type Culture Collection (ATC, Manassas, VA, USA). Cells were grown in the suggested media (Gibco Life Technologies, USA) supplemented with 10% fetal bovine serum (Gibco Life Technologies, San Diego, CA, USA) and antibiotics (Gibco Life Technologies) at 37°C under an atmosphere of 5% CO_2_. The media was changed every 3 days, and cells were passaged using 0.25% trypsin-EDTA (Gibco Life Technologies, USA) to detach cells.

### Animals

Female 6-8 weeks old specific pathogen free (SPF) BALB/c mice (average body weight of 19 ± 2 g) were used in the experiments. Mice were purchased from the Experimental Animal Center of the Chinese PLA General Hospital (PLAGH, Beijing, China) and were bred at the accredited animal housing facility under standard conditions of temperature, humidity and light/dark photoperiod. They were provided with standard mouse chow and water ad libitum and adapted to the breeding conditions for one week before use in the experiments. All animal procedures were performed with the prior approval of the Institutional Animal Ethics Committee of the Beijing Institute of Radiation Medicine (BIRM) and carried out strictly according to the AMMS Guidelines for the Care and Use of Laboratory Animals.

### Irradiation

Mice were placed in well-ventilated and perspex-covered containers and subjected to TBI at the institutional ^60^Co γ-irradiation facility. Two different dose (7.5Gy for the 20-day survival study and 10Gy for the others) were used in the experiments. The distance between the animals and the source of radiation was about 4 m and the UNIDOS® E Universal Dosemeter (PTW-Freiburg Inc, Freiburg, Germany) used to measure the dose rates. Following irradiation, mice were returned to their original cages and monitored daily.

### Flow cytometry (FCM) analysis

The apoptosis and necrosis rates of the IEC-6 cells were analyzed by FCM at 48 h after irradiation of 10Gy.

### Cell proliferation assay

Of IEC-6 cells were seeded in a 96-well cell culture 5 ×10^4^ cells/well. The effects of TPA (1 nM) on cell proliferation following radiation were assessed daily by cell counting using Cell Counting Kit-8 (Sigma, St. Louis, MO, USA).

### Cell cycle analysis

After an incubation period of 3-5 day, IEC-6 cells were harvested and treated with 70% ice-cold ethanol overnight. The cells were treated with RNase (50 μg/mL) at 37°C for 1 h and then were treated with PI (20 μg/mL) for 30 min at 4°C in the dark. The DNA content was analyzed by flow cytometry (Beckman Coulter Indianapolis, IN, USA).

### Immunohistochemistry

The paraffin sections (3μm-thick) were deparaffinized, rehydrated with graded alcohol solutions. Antigen retrieval was performed by heating the sections in a 99°C water bath for 40 minutes. After endogenous peroxidase activity was quenched and nonspecific binding was blocked, the sections were incubated with antibodies to Ki67 (Ventana Medical Systems, Tucson, Arizona, USA) at room temperature for 30 minutes. Slides were incubated with the secondary antibody (Flex HRP) for 30 minutes. After the slides were washed, they were incubated with Flex DAB chromogen and counterstained with Flex hematoxylin.

### Microcolony assay

Following the IR treatment, mice were euthanized on day 3.5. The whole intestine was fixed and distal jejunum was subjected to Ki67 immunostaining. The number of surviving crypts in the intestine was measured. A surviving crypt was defined as containing five or more adjacent, Ki67-positive nuclei [47]. The number of crypts in each circumference was recorded. Ten circumferences per mouse (0.5 to 1 cm apart) and 3 mice per experimental group were assessed [47]. All the 30 cross-sections were averaged together and represented.

### Animal survival study

Mice were exposed to TBI with the lethal dose of 7.5Gy at a dose rate of 100.2cGy/min. This dose was selected for TBI based on our empirically determined LD_50_ value. Mice were monitored for 20 days. The body weights and the number of dead (if any) mice were recorded daily. Survival curves were drawn and analyzed by Prism 6.0 software (GraphPad Inc., Carlsbad, CA, USA).

### Western blot analysis

The cells were harvested and lysed in 1% SDS on ice. The supernatant was collected, and the protein concentration was determined with the Pierce BCA Protein Assay Kit (Thermo Scientific, Waltham, MA, USA). Equivalent amounts of protein (20 μg) from each sample were separated by electrophoresis, transferred to a membrane, and incubated with the specific antibodies. The immunoblots were visualized by chemiluminescence. Actin was probed to ensure equal protein loading.

### Real-time quantitative polymerase chain reaction (qRT-PCR)

Total RNA isolated from mouse intestines (24h post-IR) (n = 3 per group and one sample per mouse) was subjected to reverse transcription with Superscript II RNase H—Reverse Transcriptase and random hexanucleotide primers. The RNA samples were assessed for quantification and purity by A260/A280 absorption, and RNA samples with ratios greater than 1.8 were stored at −80°C for further analysis. qRT-PCR was performed using a MMLV-Reverse Transcriptase kit (EPICENTRE® Biotechnologies, Madison, WI, USA). Complementary DNA (cDNA) was then synthesized. Information for primers used in qRT-PCR was described in Supplementary Table 1.

### Statistical analysis

Student *t*-test and one-way ANOVA were used to analyze the *in-vitro* and *in-vivo* data using Prism the 6.0 software (GraphPad Inc.). The Kaplan–Meier method and the log-rank test were used to compare the overall survival, and were also conducted with statistical analysis software program Prism 6.0 software (GraphPad Inc.). *P < 0.05, **P < 0.01, ***P < 0.001, ****P < 0.0001, as compared with the control/normal group.

## SUPPLEMENTARY TABLE


