# Supplementary material for: 12-O-tetradecanoylphorbol-13-acetate (TPA) increases murine intestinal crypt stem cell survival following radiation injury
Source: Oncotarget. 2017 Apr 20;8(28):45566–76. doi: 10.18632/oncotarget.17269 (PMC5542208; doi:10.18632/oncotarget.17269)
Supplement: Supplementary file 1 [file oncotarget-08-45566-s001.pdf]

12-*O*-tetradecanoylphorbol-13-acetate (TPA) increases murine intestinal crypt stem cell survival following radiation injury

SUPPLEMENTARY TABLE

Supplementary Table 1: Sequences of primers used in this study

β-actin	forward	5'-GGTGATCCACATCTGCTGGAA-3'
	reverse	5'-ATCATTGCTCCTCCTCAGGG-3'
Dclk1	forward	5'-CAGCAACCAGGAATGTATTGGA-3'
	reverse	5'-CTCAACTCGGAATCGGAAGACT-3'
Msi1	forward	5'-CAGTTTCGGACCTATCTCTGAGGT-3'
	reverse	5'-AAGGTGATGAAACCAAAACCCCT-3'
Notch1	forward	5'-CGGGTCCACCAGTTTGAATG-3'
	reverse	5'-GTTGTATTGGTTCGGCACCAT-3'
Lgr5	forward	5'-CAGTTTCGGACCTATCTCTGAGGT-3'
	reverse	5'-AAGGTGATGAAACCAAAACCCCT-3'
Bmi1	forward	5'-CGGGTCCACCAGTTTGAATG-3'
	reverse	5'-GTTGTATTGGTTCGGCACCAT-3'
